# Invasive Lobular Breast Carcinoma Can Be a Challenging Diagnosis Without the Use of Tumor Markers

**DOI:** 10.7759/cureus.8376

**Published:** 2020-05-31

**Authors:** Linda C Klumpp, Rony Shah, Naeem Syed, Gustavo Fonseca, Jeffrey Jordan

**Affiliations:** 1 Internal Medicine, HCA Citrus Memorial Hospital, Inverness, USA; 2 Hematology and Oncology, HCA Citrus Memorial Hospital, Inverness, USA; 3 Hematology and Oncology, Florida Cancer Specialists, Lecanto, USA

**Keywords:** invasive lobular carcinoma, e-cadherin, tumor markers, mammogram, er positive, her 2 negative, metastatic breast cancer, cam 5.2, single-file pattern, hematoxylin and eosin

## Abstract

Invasive lobular carcinoma is often challenging to diagnose due to the lack of physical examination findings and macrocalcifications on mammography. The cells of invasive lobular carcinoma form a distinct single file pattern that can be identified on histology slides. Often, when patients present, there is metastasis to the bones, lymph nodes, and gastrointestinal tract. Tumor markers are a valuable tool in identification, especially the loss of E-cadherin protein. However, if E-cadherin protein is not available, epidermal membrane antigen, which inhibits E-cadherin, can prove to be a significant diagnostic tool. Epidermal membrane antigen was the key tumor marker in our patient case. Other tumor markers and histology stains can drive treatment plans and help predict prognosis.

## Introduction

Invasive lobular carcinoma (ILC) comprises 10-15% of invasive breast cancers. It is far less common than invasive ductal breast cancer, which makes up approximately 80% of cases [1]. Diagnosis can be difficult to detect through diagnostic imaging due to the lack of architectural distortion and macrocalcifications on mammography [1]. Thus, this gives a high chance for metastasis and delays diagnosis. ILC is distinguished from intraductal invasive carcinoma by its tendency, with malignant cells forming a single file in the mammary ducts and lobules on histopathology [2]. ILC can frequently metastasize to sites that include the gastrointestinal tract, the peritoneum, lymph nodes, and the adnexa [3]. At the time of diagnosis, two-thirds of patients have metastases. Tissue biopsy with tumor markers may prove to be the most beneficial diagnostic tool. Many studies have associated ILC with has a poor outcome when compared with other forms of invasive carcinoma. The National Cancer Institute reports a 22% five-year survival rate [4].

## Case presentation

We present a case of a 54-year-old female, with a medical history of hypertension and hypothyroidism who presented to the emergency room with severe weakness and anemia. The patient presented twice in a one-month period. On the first occasion, our patient presented with nausea and flank pain. She was diagnosed and treated for urinary tract infection and nephrolithiasis. At that time, she was found to be anemic, with a hemoglobin of 8.6 gm/dL and hematocrit of 24.3 gm/dL. She was asymptomatic at the time. The patient returned to the emergency room a few weeks later with the complaint of severe weakness, shortness of breath, decreased appetite, and weight loss of 12 pounds since prior visit. Pertinent history included a total hysterectomy secondary to menorrhagia and former tobacco use (24 pack-year history). Maternal history included terminal ovarian cancer, which was diagnosed around age 60. Bilateral mammogram at age 45 was negative.

The patient was ill-appearing, with pale conjunctiva and skin. On examination, she was hypertensive with a blood pressure of 194/110 mmHg and tachycardic with a heart rate of 108 bpm. Tenderness to palpation was noted over the lumbar spine. Laboratory results were as follows: sodium level of 131 mEq/L, potassium level of 4.0 mEq/L, BUN (blood urea nitrogen) level of 18 mg/dL, creatinine level of 1.1 mg/dL, white blood cell count of 14.5 x 103/mL, hemoglobin level of 7.1 gm/dL, hematocrit of 21.4 gm/dL, platelet count of 57 x 103/mL, MCV (mean corpuscular volume) of 108.5 fL, MCH (mean corpuscular hemoglobin) of 35.3 pg, RDW SD (red cell distribution width standard deviation) of 104.3 fL, RDW CV (red cell distribution width coefficient of variation) of 29.5 fL, nucleated red blood cell (RBC) of 9.0%, absolute lymphocyte count of 4.96 x 103/µL, RBC count of 2.01 x 106/µL, and rare smudge cells on blood smear.

**Figure 1:**
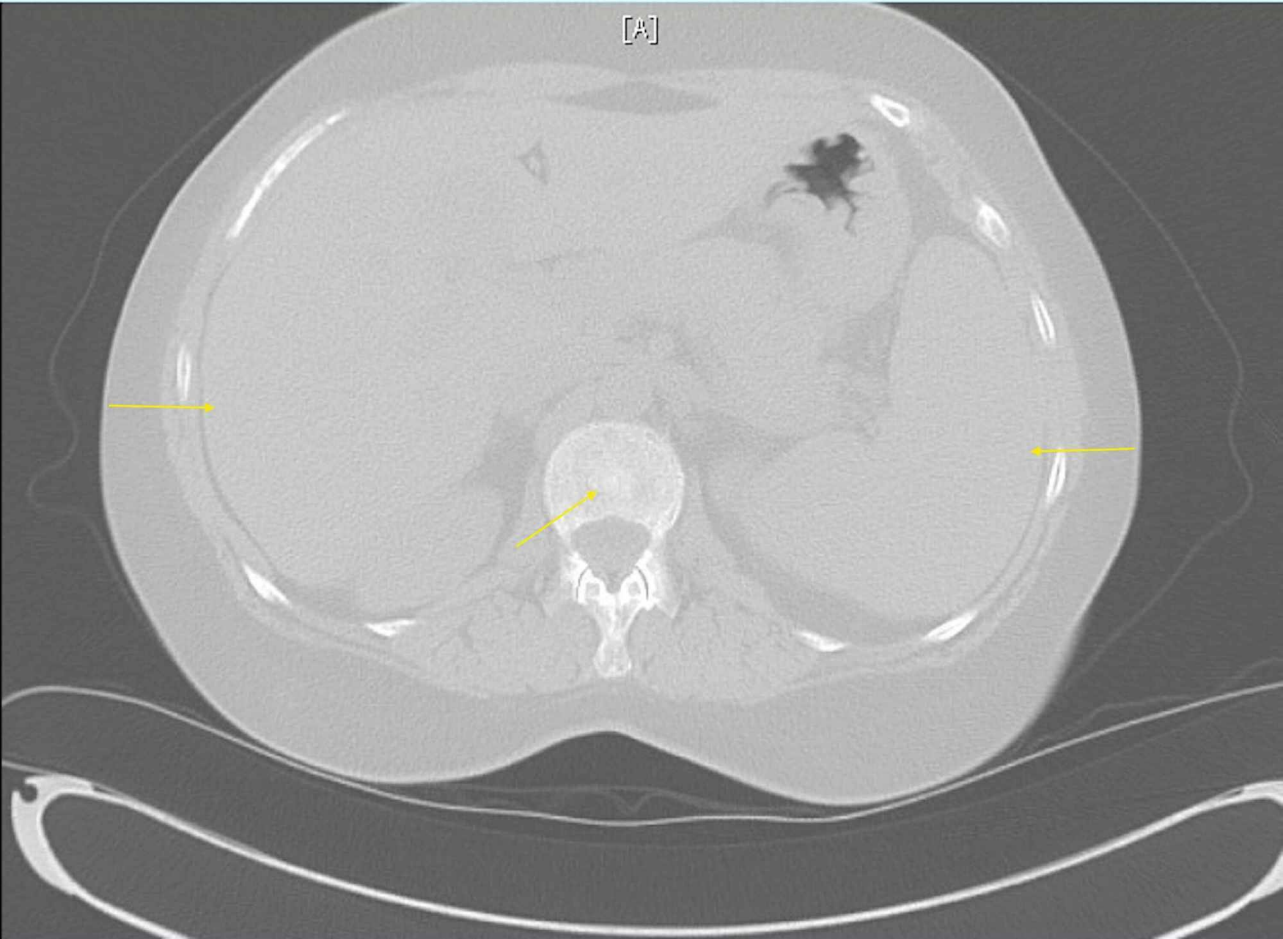
Figure 1: CT of the abdomen/pelvis without contrast Hepatosplenomegaly and patchy sclerosis of bones can be seen.

**Figure 2:**
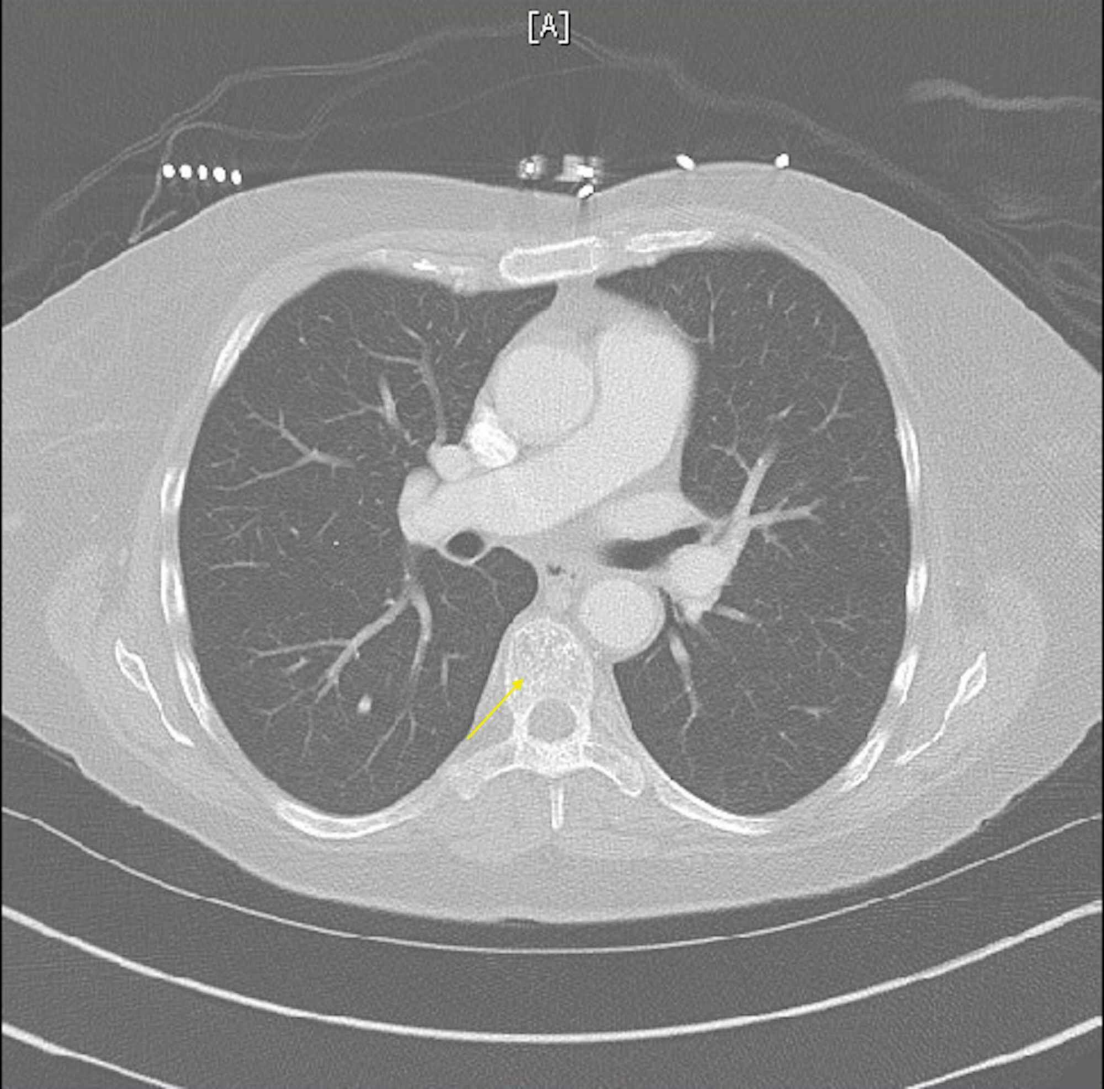
CT of the chest without contrast Diffuse lytic and sclerotic bone lesions can be seen.

The patient was admitted to the hospital for further workup of anemia. Computed tomography (CT) of the abdomen/pelvis with contrast was reviewed and non-obstructing calculi were identified in the upper pole of the left kidney; also noted were areas of patchy sclerosis of the bones, possibly secondary to osteoporosis or infiltrative process such as leukemia (Figure 1). Hepatomegaly and splenomegaly were also noted (Figure 1). CT of the chest with contrast in comparison with CT of the abdomen/pelvis revealed diffuse sclerotic and lytic lesions throughout (Figure 2). Findings were suggestive of either a metastatic disease or myeloma. The Hematology-Oncology team was consulted for anemia and thrombocytopenia. Hematology test ordered included beta-2 microglobulin, direct antiglobulin test (DAT), lactate dehydrogenase (LDH), methylmalonic acid, reticulocyte count, and uric acid. Uric acid level was elevated, and treatment with allopurinol was initiated.

**Figure 3:**
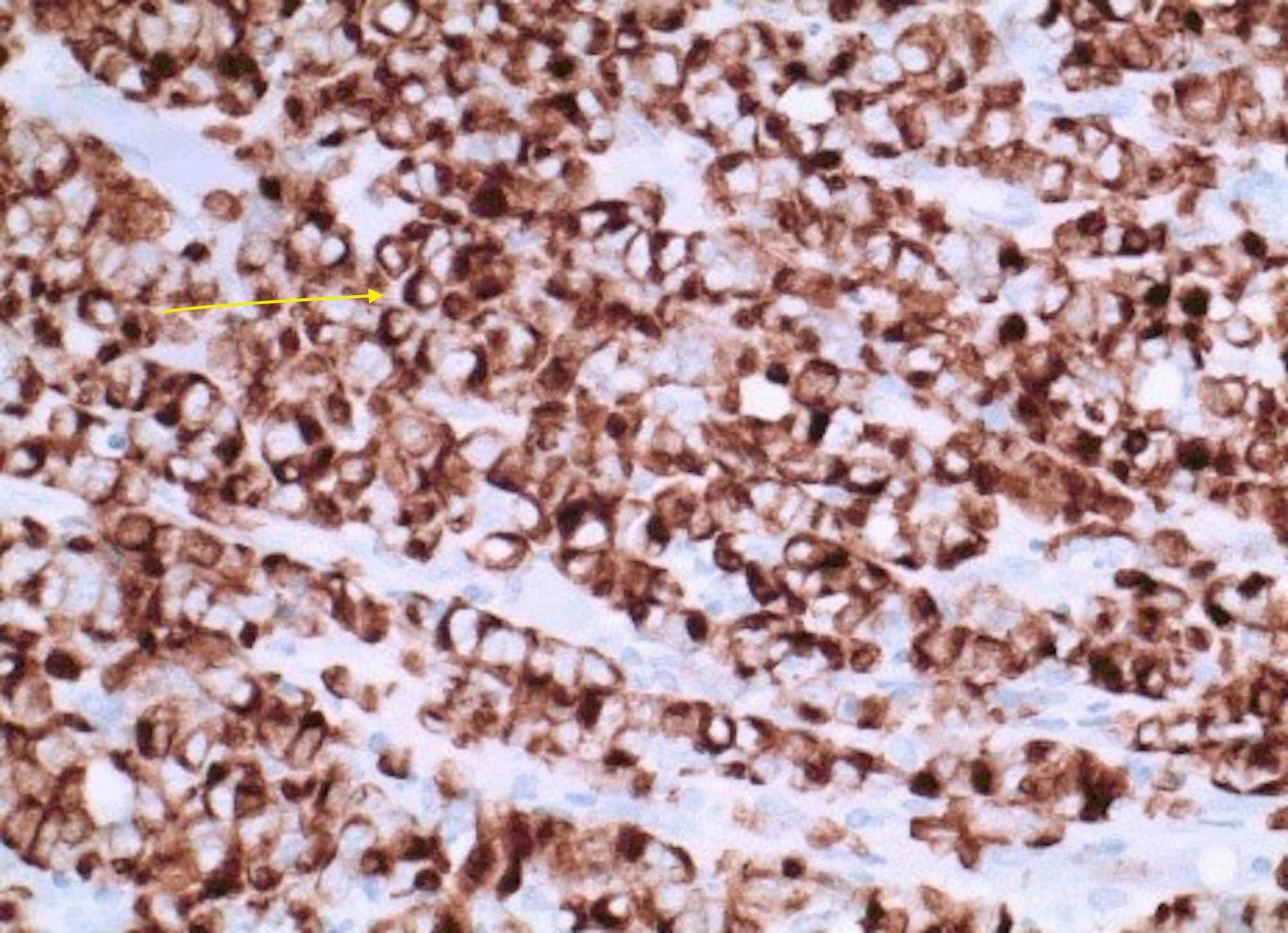
Anti-cytokeratin reagent stain (CAM 5.2)

**Figure 4:**
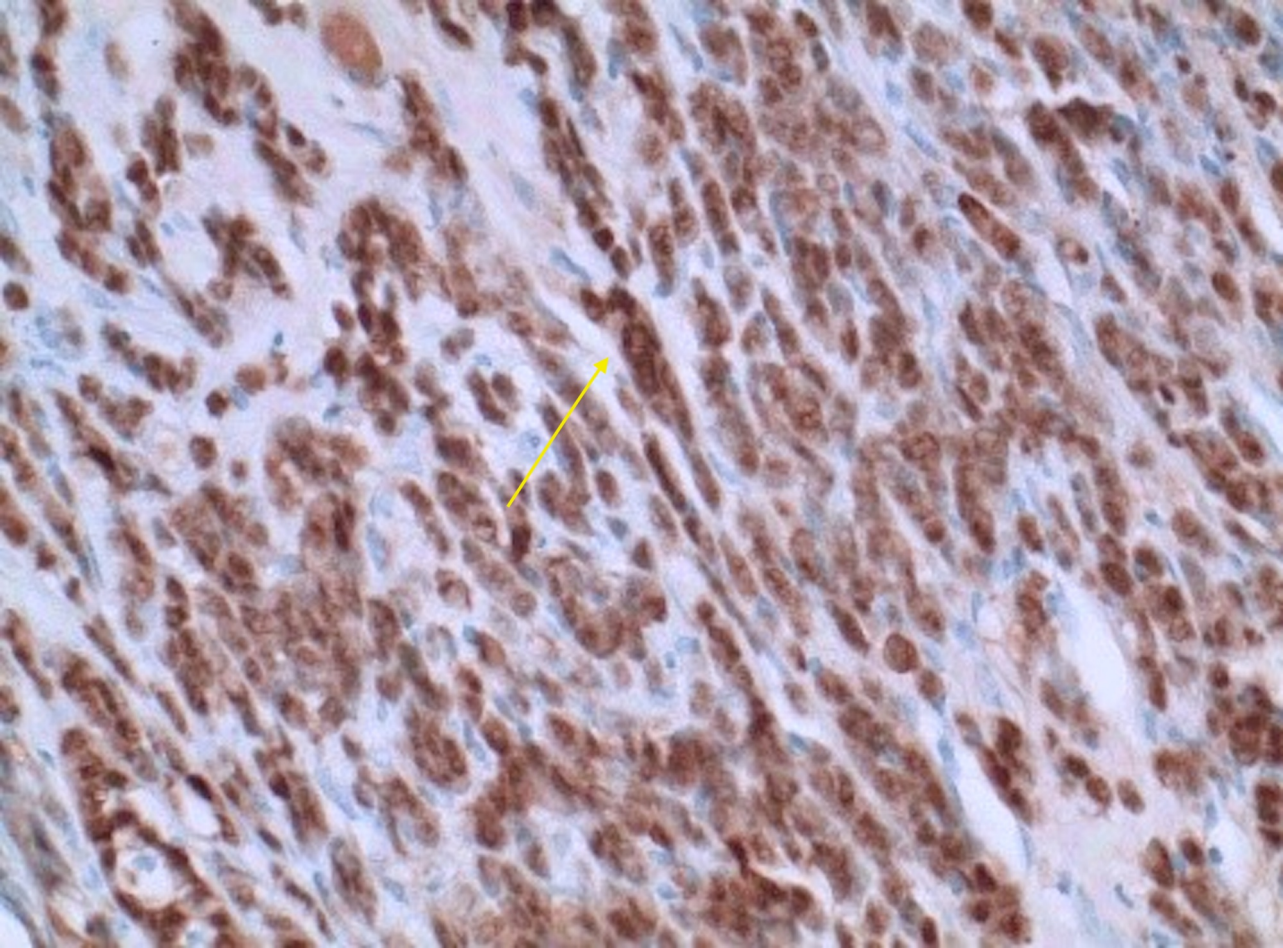
ER marker for lobular breast adenocarcinoma ER, estrogen receptor

**Figure 5:**
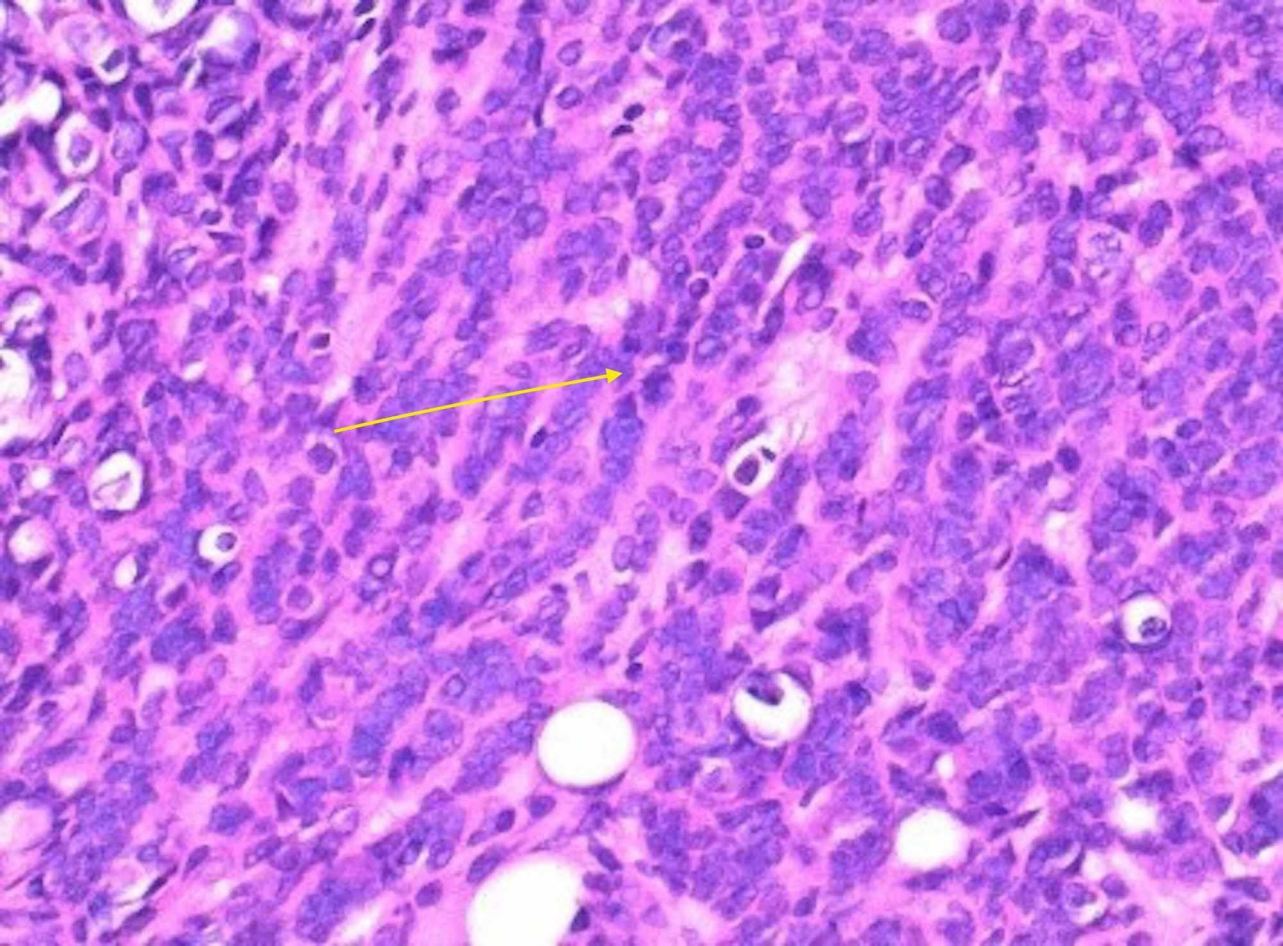
H&E stain H&E, hematoxylin and eosin

A total of four bone marrow biopsies were performed at the bedside and with CT guidance. Samples were drawn from the iliac crests. All biopsies resulted in a dry tap. The dry taps further raised suspicion for acute lymphocytic leukemia or lymphoma. Tumor markers and histology stains were sent out for further analysis (Table 1) (Figures 3-5).

**Table 1:** Tumor Marker and Histology Stains EMA, epithelial membrane antigen; WT1, Wilms tumor 1; ER, estrogen receptor; TTF1, thyroid transcription factor 1; CDX2, caudal type homeobox 2; CK20, cytokeratin 20; HER2, human epidermal growth factor 2

EMA (marker for metastatic carcinoma): strongly positive
WT1 gene (gene expressed in leukemia): negative
Anti-cytokeratin reagent stain (CAM 5.2) (Figure 3)
ER (marker for metastatic lobular adenocarcinoma from breast): strongly positive (Figure 4)
Hematoxylin and eosin stain (Figure 5)
TTF1 (sensitive marker for pulmonary and thyroid adenocarcinoma): negative
CDX2 (marker for gastrointestinal cancer): negative
CK20 (marker for colorectal carcinoma): negative
HER2 (protein expressed on breast cells): negative

**Figure 6:**
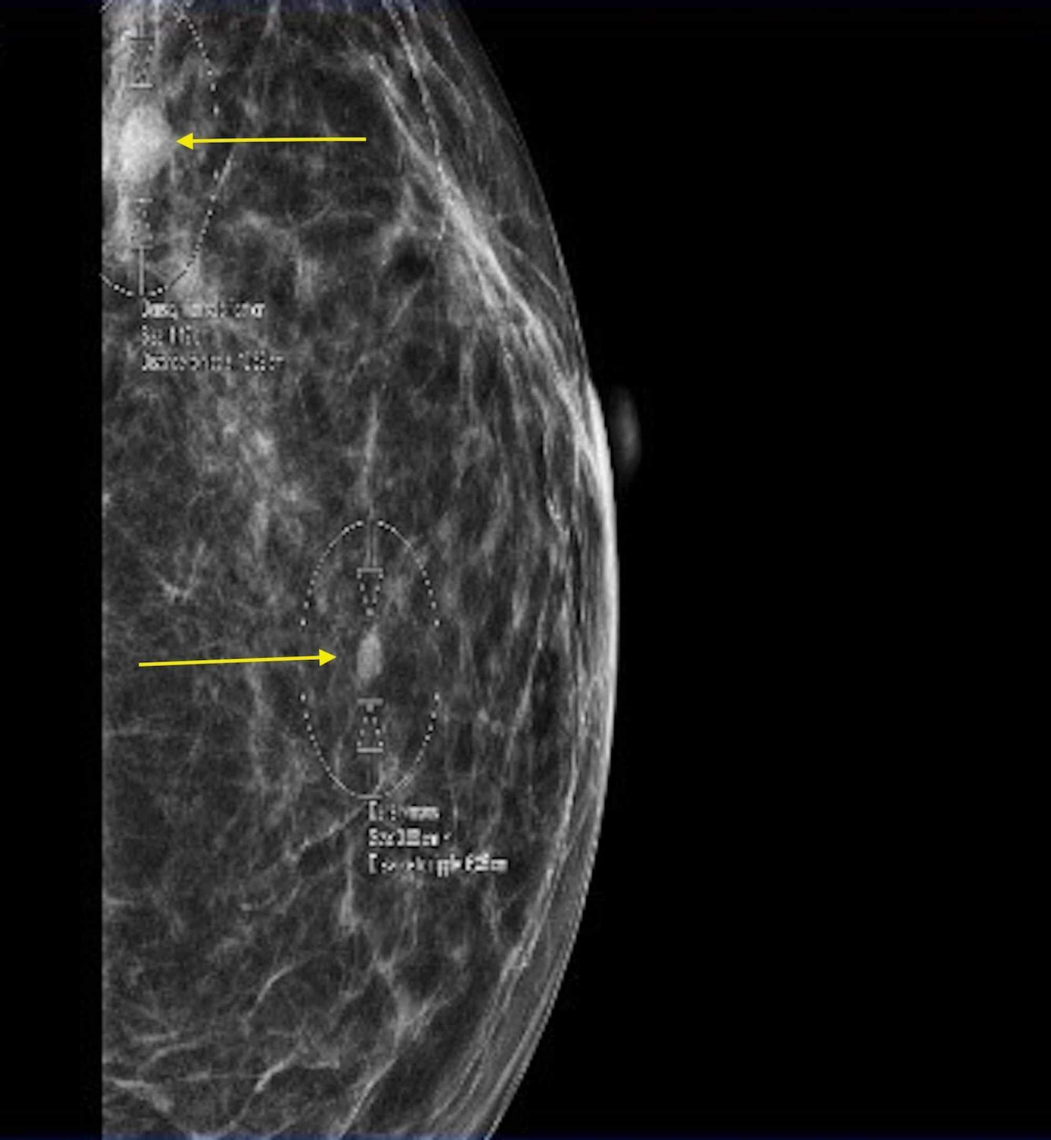
Mammogram of the left breast Pleomorphic calcification with a 1-cm well-circumscribed mass in the left upper outer breast (top arrow) can be seen.

**Figure 7:**
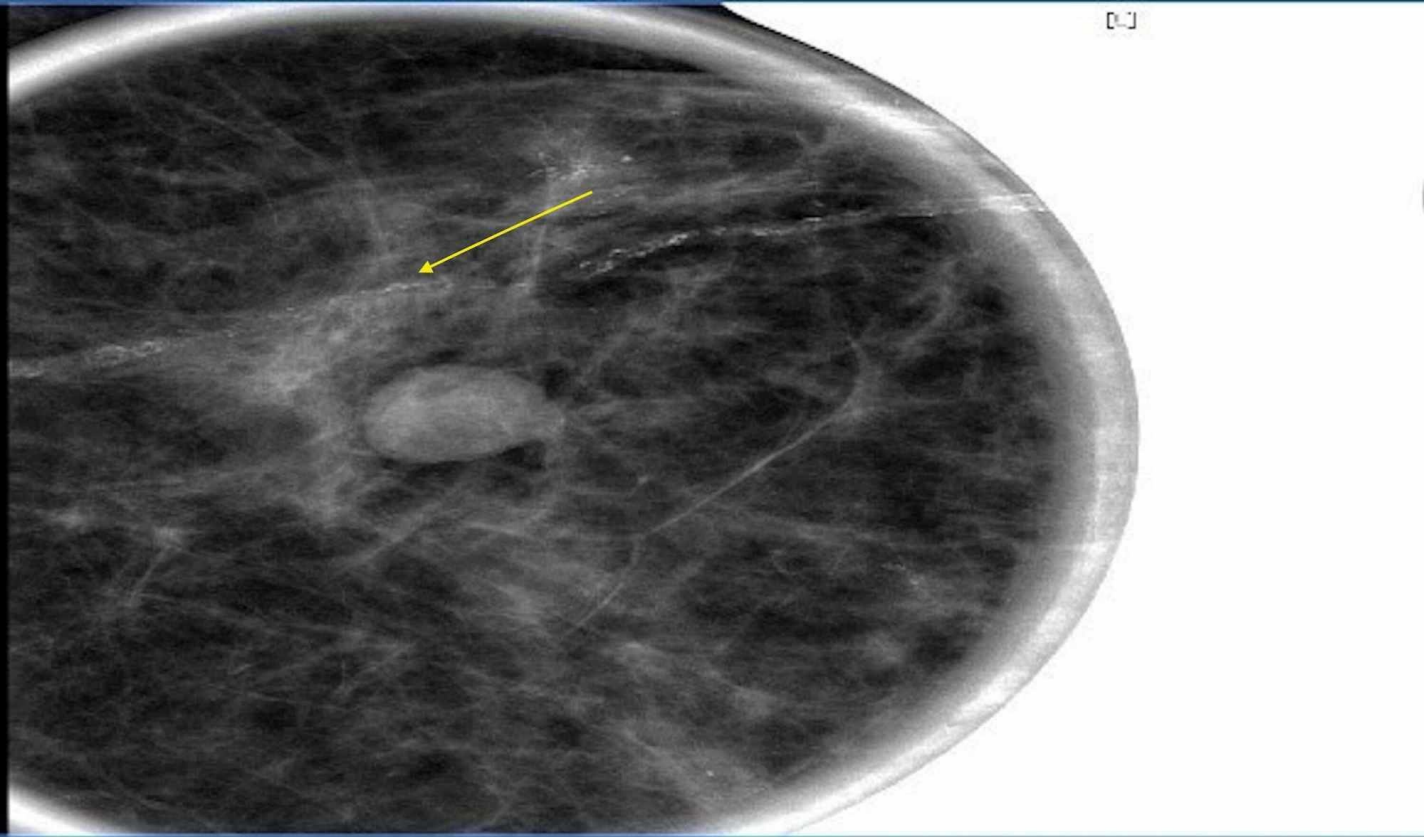
: Mammogram of the left breast Left breast architectural distortion and single-file microcalcifications can be seen.

**Figure 8:**
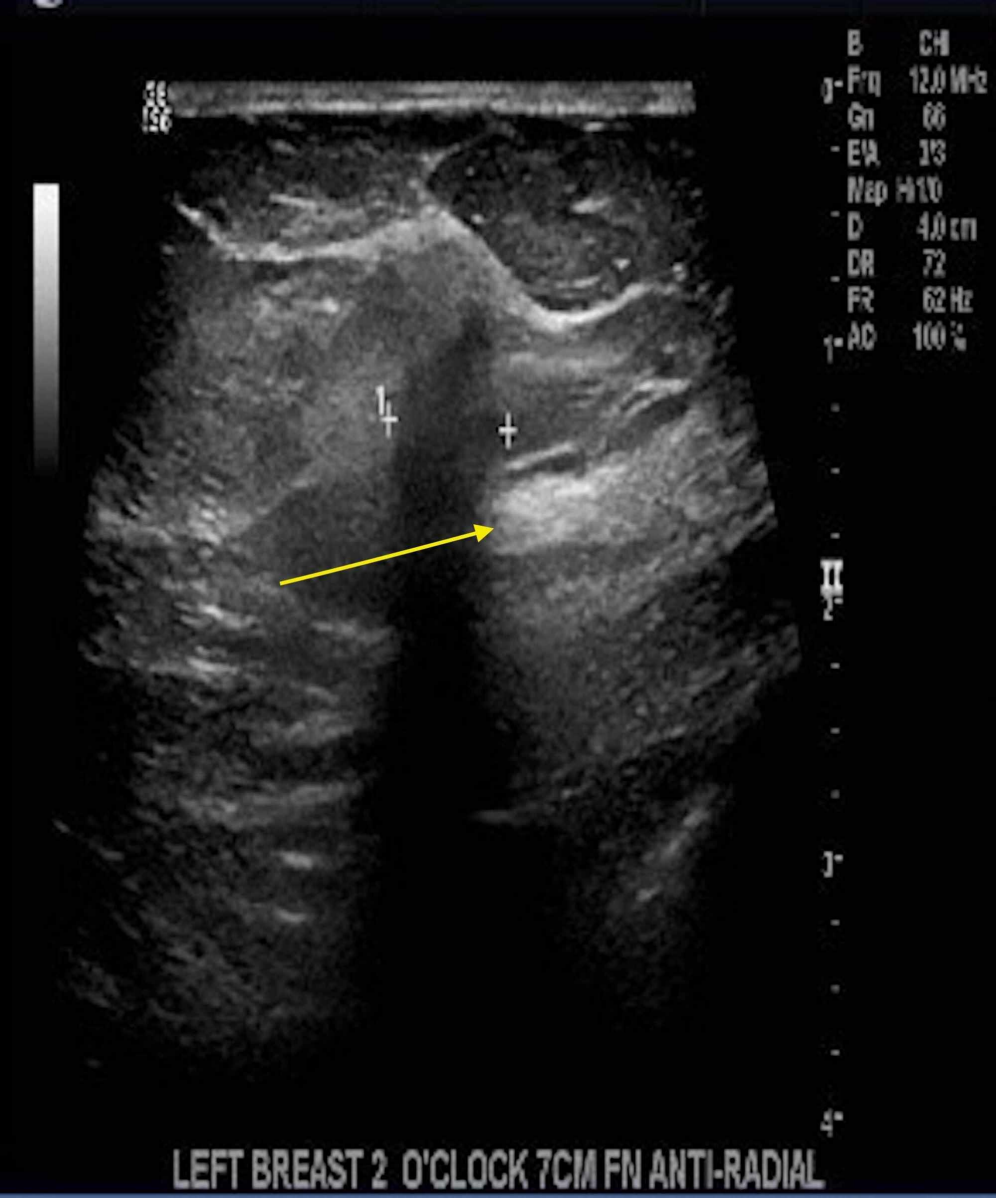
Ultrasound of the left breast A 4-mm hypoechoic nodule at the 3’o clock position, 4 cm from the nipple, can be seen.

A mammogram and bilateral breast ultrasound performed further reinforced the diagnosis of lobular breast cancer. The mammogram findings showed scattered fibroglandular densities. Pleomorphic calcifications were present in the upper outer left breast, and a 1-cm well-circumscribed mass was seen in the upper outer left breast (Figure 6). In the left breast, there were architectural distortion and single-file microcalcifications (Figure 7) [5]. The ultrasound findings showed a shadowing mass at the 2 o'clock position, 7 cm from the nipple, which likely corresponded to pleomorphic calcifications seen on mammogram. A 4-mm hypoechoic nodule was seen at the 3 o'clock position, 4 cm from the nipple (Figure 8). Imaging further coincided with the diagnosis of lobular breast cancer.

The patient was discharged and referred to outpatient follow-up to start treatment with letrozole, palbociclib, and transfusions, as needed.

## Discussion

ILC is the second most common type of invasive breast malignancy and comprises 10-15% of cases [6]. ILC has a distinct metastatic pattern with spread to the bones and gastrointestinal tract [6]. ILC has a high false-negative rate in screening with imaging and physical examination due to its morphology, and thus often it presents later in the disease course with metastasis. The risk for ILC is strongly associated with early menarche, late menopause, and late age at first birth. The epithelial membrane antigen (EMA) marker for metastatic breast cancer is significant because this particular antigen inhibits E-cadherin. In our patient, EMA was strongly positive. The CDH1 gene, the gene which codes for the E-cadherin adhesion protein, is of special interest as mutations are associated with ILC, but never with ductal carcinoma. The excess of breast cancers of the lobular type in CDH1 families led researchers to identify it also as a susceptibility gene for ILC [7]. Loss of E-cadherin is common and supports the diagnosis of ILC. Estrogen receptor positive (ER+) and human epidermal growth factor negative (HER-) receptors aid in diagnosing the disease and predicting treatment. ER positive is consistent with luminal mammary breast cancer. HER2- is associated with breast, bladder, ovarian, pancreatic, and stomach cancers. Treatment can be directed at hormone therapy with ER+ and HER2-.

Based on research, breast-specific gamma imaging (BSGI) has the highest sensitivity at 93% for the detection of ILC [8]. The sensitivities of mammography, sonography, and MRI were 79%, 68%, and 83%, respectively [8]. BSGI is an effective technique and should be used in diagnosis.

Treatment with a selective inhibitor of cyclin-dependent kinases (CDK4 and CDK6) such as palbociclib has been proven to be effective in preventing cancer cells from entering into the G1 phase of the cell cycle. Palbociclib prevents the phosphorylation of retinoblastoma protein, which prevents cancer cells from passing a checkpoint in the cell cycle and proceeding to divide. Aromatase inhibitors inhibit estrogen, thus suppressing the recurrence of the breast tumor tissue. ILC can be treated with lumpectomy or mastectomy if diagnosed in the early stages [9]. The survival rate of women with stage IV ILC is 22% [10]. The combination of CDK inhibitors and an aromatase inhibitor can increase progression-free survival rate by up to 24.8 months.

## Conclusions

Although ILC is common, the diagnosis can be difficult based on common imaging modalities and screening practices. ILC does not form a lump and spreads through fatty breast tissue in a single file. Unexplained bone pain, anemia, and gastrointestinal disturbances should raise physician suspicion in the absence of other obvious causes. Although our patient presented as having a possible hematological or possible lymphocytic malignancy in nature, tumor markers helped in making a positive diagnosis of metastatic lobular breast carcinoma. This indicates the importance of the role that tumor markers play in making a definitive diagnosis of ILC.
